# Base-First Versus Tip-First Appendicectomy: A Retrospective Analysis of Surgical Technique, Pathological Yield, and Oncological Safety

**DOI:** 10.7759/cureus.93073

**Published:** 2025-09-23

**Authors:** Kapil Agrawal, Jaspreet Singh Kaur, Ravi Chandavarkar, Shumaila Tanveer, Ben Liu

**Affiliations:** 1 General Surgery, The Royal Wolverhampton NHS Trust, Wolverhampton, GBR; 2 General Surgery, New Cross Hospital, Wolverhampton, GBR; 3 Colorectal Surgery, The Royal Wolverhampton NHS Trust, Wolverhampton, GBR

**Keywords:** appendiceal neoplasm, histology post appendicectomy, laparascopic appendicectomy, mesoappendix, oncological safety, surgical technique of appendix

## Abstract

Background: Appendicectomy is a common emergency operation, but there is no consensus on the optimal operative technique. We defined and compared two approaches: the tip-first technique and the base-first technique. In the tip-first technique, dissection begins at the distal appendix and progresses toward the base, stripping the mesoappendix off the appendix and leaving it behind. In the base-first technique, dissection starts near the base of the appendix with division of the appendiceal artery near its origin, removing the appendix together with its mesoappendix. Because the mesoappendix contains lymphovascular structures essential for pathological assessment and oncological staging, its omission may compromise completeness. This study aimed to classify appendicectomy into tip-first and base-first techniques and compare their clinical, operative, and pathological outcomes. We hypothesised that the base-first technique would consistently include the mesoappendix, ensuring more complete staging.

Methods: A retrospective cohort study was conducted of 134 consecutive appendicectomies performed at a single United Kingdom institution from January to December 2022. Techniques were classified histologically as base-first (mesoappendix present) or tip-first (mesoappendix absent). Data collected included demographics, American Society of Anesthesiologists (ASA) grade, surgeon grade, operative details, specimen characteristics, incidental malignancies, and clinical outcomes. Statistical analysis used chi-square, t-tests, and Mann-Whitney U tests, with p<0.05 considered significant.

Results: Of 134 patients, 94 (70.1%) underwent base-first and 40 (29.9%) tip-first appendicectomy. Demographics were comparable, although base-first patients were slightly older (mean 40.5 vs. 38.3 years; Student’s t-test (t(132)=1.97), p=0.051). Consultant-led surgeries were more frequent in the base-first group (41.5% vs. 22.5%; chi-square test (χ²(1)=4.39), p=0.036). Median length of stay was similar (four vs. three days; Mann-Whitney U=1763, p=0.589). Incidental neoplasms were found in five patients: three (3.2%) in the base-first group and two (5.0%) in the tip-first group (χ²(1)=0.23, p=0.633). In tip-first cases, lack of mesoappendix hindered staging, requiring further right hemicolectomy.

Conclusion: This is, to our knowledge, is the first study to categorise appendicectomy techniques into tip-first and base-first groups with comparative outcomes. While overall clinical results were comparable, tip-first appendicectomy risks incomplete oncological staging due to the absence of the mesoappendix, occasionally necessitating further major surgery. Surgical training should emphasise the base-first technique to ensure pathological completeness and oncological safety.

## Introduction

Acute appendicitis is one of the most common surgical emergencies worldwide, with a lifetime risk of 7%-8% in Western populations [1,2]. Appendicectomy remains the definitive treatment, and it is among the most frequently performed emergency abdominal procedures [3]. The introduction of laparoscopy revolutionised appendicectomy, reducing postoperative pain, hospital stay, and wound infection compared with open surgery [4,5]. However, beyond the choice of open versus laparoscopic approach, there are further technical variations that remain underexplored.

One such variation concerns the handling of the mesoappendix. We defined two distinct approaches. In the tip-first technique, dissection begins at the distal appendix and progresses toward the base, stripping the mesoappendix away from the appendix and leaving it behind. In the base-first technique, dissection starts near the base of the appendix with division of the appendiceal artery close to its origin, enabling removal of the appendix together with its mesoappendix.

Surgeons may choose the tip-first technique because it can seem technically easier, reduce immediate bleeding from the mesoappendix, or save operative time. However, the mesoappendix contains the appendiceal artery and lymphovascular structures critical for pathological staging when incidental neoplasms are identified. If the mesoappendix is not removed, pathological staging may be incomplete, sometimes requiring more extensive surgery such as right hemicolectomy [6-8].

Despite appendicectomy being a cornerstone of surgical training, variation in technique persists. This likely reflects historical practices, differences in surgeon training, absence of consensus recommendations, and a limited evidence base comparing outcomes of these approaches [9-11].

We therefore conducted a retrospective cohort study to classify appendicectomy techniques into base-first and tip-first approaches and to compare their clinical, operative, and pathological outcomes. We hypothesised that the base-first technique would consistently include the mesoappendix, thereby ensuring more complete oncological staging compared with the tip-first technique.

## Materials and methods

This was a single-centre retrospective cohort study conducted at The Royal Wolverhampton NHS Trust in Wolverhampton, United Kingdom. All consecutive adult patients (≥16 years) undergoing laparoscopic appendicectomy between 1 January 2022 and 31 December 2022 were eligible for inclusion. Patients who underwent open or interval appendicectomy after non-operative management, as well as those with incomplete records, were excluded.

Patients were grouped according to the histopathological appearance of their specimens. The base-first technique group consisted of cases where the mesoappendix was present, while the tip-first technique group consisted of cases where the mesoappendix was absent.

Data were collected from electronic patient records and pathology reports into a standardised database. Two investigators independently performed data extraction, and discrepancies were resolved by consensus. Inter-rater reliability was not formally assessed and is acknowledged as a limitation.

The variables collected included age, sex, American Society of Anesthesiologists (ASA) grade, urgency of surgery (emergency or elective), operative approach (laparoscopic or open), operative duration, and operating surgeon grade (consultant or trainee). Pathological variables included appendix length, mesoappendix length, and the presence and histological subtype of incidental malignancy. Clinical outcomes included length of stay; postoperative complications defined as surgical site infection, intra-abdominal abscess, postoperative ileus, or 30-day readmission, and the need for further surgery prompted by pathological findings.

All specimens were examined by consultant histopathologists according to institutional practice. Representative intraoperative and specimen images were obtained to illustrate the differences between appendicectomy techniques: base-first (appendix with mesoappendix present) and tip-first (appendix without mesoappendix).

The study protocol was reviewed and approved by the Institutional Review Board of The Royal Wolverhampton NHS Trust. All data were anonymised and de-identified prior to analysis. The study was conducted in accordance with the Declaration of Helsinki.

Statistical analysis was performed using IBM SPSS Statistics software, version 27 (IBM Corp., Armonk, NY, USA). Continuous variables were tested for normality and are reported as mean with standard deviation for normally distributed data or as median with interquartile range for non-normal data. Normally distributed continuous variables were compared using the Student’s t-test, non-normally distributed data with the Mann-Whitney U test, and categorical variables with chi-square or Fisher’s exact test as appropriate. A two-tailed p<0.05 was considered statistically significant.

## Results

A total of 134 patients underwent appendicectomy during the study period, comprising 94 (70.1%) procedures performed using the base-first technique and 40 (29.9%) using the tip-first technique. The baseline demographic characteristics of the two groups were broadly comparable (Table 1). Patients in the base-first cohort were slightly older than those in the tip-first group (mean age 40.5 years vs. 38.3 years), although this difference did not reach statistical significance (Student’s t-test, t(132)=1.97, p=0.051). The gender distribution was similar between groups, with females representing 49.5% of the base-first group and 40.0% of the tip-first group (chi-square test, χ²(1)=1.01, p=0.314). Correspondingly, the proportion of males was 50.5% in the base-first group and 60.0% in the tip-first group. Distribution of ASA grades was also comparable between the two groups (χ²(3)=1.64, p=0.650).

**Table 1:** Comparison of patient demographics, operative characteristics, and pathological outcomes between base-first and tip-first appendicectomy ASA: American Society of Anesthesiologists; mm: millimetres; d: days; t: Student’s t-test statistic; χ²: chi-square statistic; U: Mann–Whitney U statistic

Characteristic	Base-First (n=94)	Tip-First (n=40)	Test Statistic	p-value
Female (%)	47 (49.5)	16 (40.0)	χ²(1)=1.01	0.314
Male (%)	47 (50.5)	24 (60.0)	χ²(1)=1.01	0.314
Age (mean, years)	40.5	38.3	t(132)=1.97	0.051
Length of stay (median, d)	4	3	U=1763	0.589
ASA distribution (I–IV)	see below	see below	χ²(3)=1.64	0.650
ASA I (%)	40 (42.5)	15 (37.5)	—	—
ASA II (%)	35 (37.2)	13 (32.5)	—	—
ASA III (%)	18 (19.1)	11 (27.5)	—	—
ASA IV (%)	1 (1.1)	1 (2.5)	—	—
Emergency surgery (%)	81 (85.3)	34 (85.0)	χ²(1)=0.001	0.969
Operative duration (mean ± SD, min)	116.8 ± 67.1	117.3 ± 77.0	t(132)=0.03	0.976
Surgical approach (laparoscopic, %)	92 (97.9)	39 (97.5)	χ²(1)=0.01	0.931
Malignancy present (%)	3 (3.2)	2 (5.0)	χ²(1)=0.23	0.633
Appendix length (mm)	60.6	57.7	t(132)=0.71	0.478
Mesoappendix length (mm)	17.7	0	U=0.0	<0.001
Consultant-led (%)	39 (41.5)	9 (22.5)	χ²(1)=4.39	0.036

**Figure 1:**
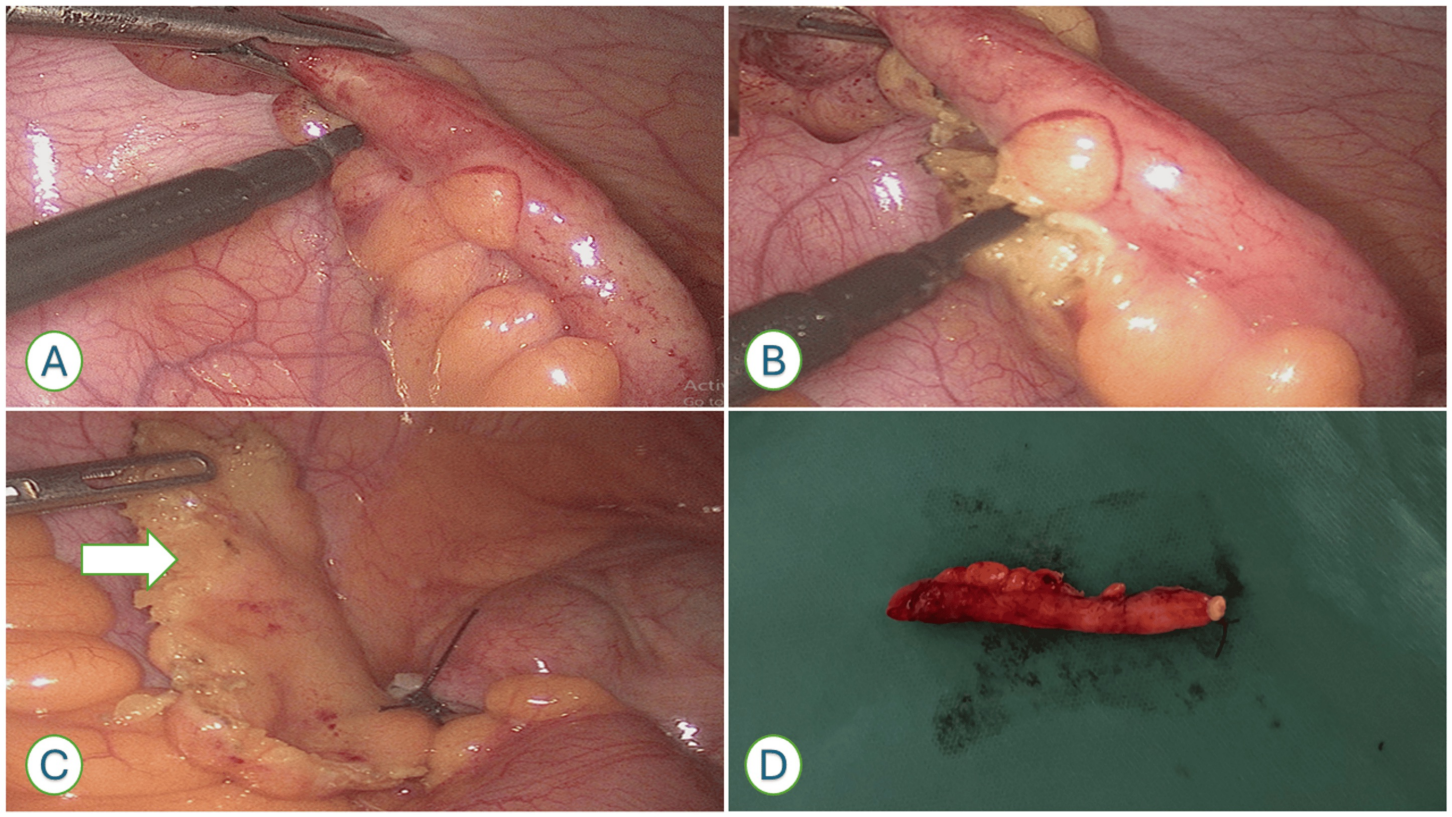
Intraoperative and specimen photographs demonstrating the tip-first appendicectomy technique (A) Intraoperative image showing dissection initiated at the distal appendix tip. (B) Progressive dissection of the mesoappendix along the appendix, stripping it away during mobilisation. (C) Completion of dissection with the appendix removed, leaving behind the mesoappendix in situ. (D) Resected specimen demonstrating the appendix without mesoappendix.

**Figure 2:**
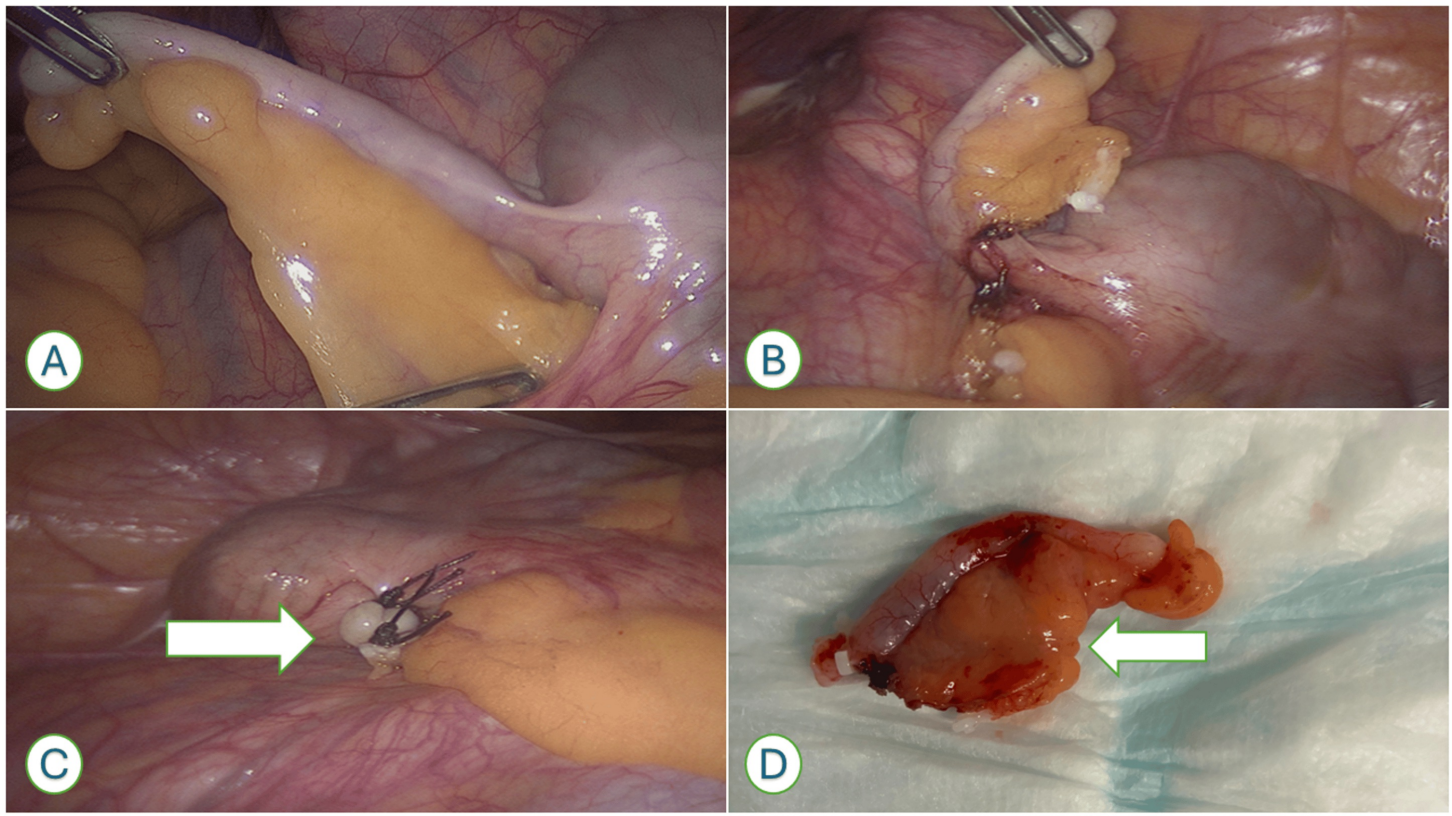
Intraoperative and specimen photographs demonstrating the base-first appendicectomy technique (A) Intraoperative image showing dissection initiated at the proximal appendix near the appendiceal artery. (B) Appendiceal artery identified and secured. (C) Appendix has been removed along with mesoappendix. An arrow highlighting the stump without the mesoappendix. (D) Resected specimen demonstrating the appendix with mesoappendix.

Operative characteristics are outlined in Table 1. Consultant-led procedures were significantly more frequent in the base-first group compared with the tip-first group (41.5% vs. 22.5%; χ²(1)=4.39, p=0.036). The majority of operations were performed as emergencies, with no significant difference in urgency between the groups (χ²(1)=0.001, p=0.969). Operative duration and surgical approach (laparoscopic versus open) did not differ significantly and have been included in Table 1. Representative intraoperative and specimen images illustrating both base-first and tip-first appendicectomy are shown in Figures 1, 2.

Pathological findings demonstrated clear differences between the two approaches. All base-first specimens consistently included the mesoappendix, with a mean mesoappendix length of 17.7 millimetres (mm). In contrast, none of the tip-first specimens included the mesoappendix (Mann-Whitney U test, U=0.0, p<0.001). The mean appendix length was slightly longer in the base-first group (60.6 mm) compared with the tip-first group (57.7 mm), although this difference was not statistically significant (t(132)=0.71, p = 0.478).

Incidental appendiceal malignancies were identified in five patients overall, corresponding to an incidence of 3.7%. Three of these cases (3.2%) occurred in the base-first group, while two cases (5.0%) were identified in the tip-first group (χ²(1)=0.23, p=0.633). The histological subtypes included adenocarcinoma (n=2), neuroendocrine tumour (n=2), and goblet cell adenocarcinoma (n=1). Staging data were available for three cases. Importantly, in both tip-first malignancy cases, the absence of the mesoappendix precluded complete oncological staging, leading to the need for a complete right hemicolectomy.

Clinical outcomes were comparable between the two techniques. The median postoperative length of stay was four days for patients undergoing base-first appendicectomy and three days for those treated with the tip-first approach (Mann-Whitney U test, U=1763, p=0.589). Tracked postoperative complications included surgical site infection, intra-abdominal abscess, postoperative ileus, and 30-day readmission. No significant differences were observed in complication rates between the two groups.

## Discussion

This study is, to our knowledge, the first to formally categorise appendicectomy into base-first and tip-first techniques and compare their clinical, operative, and pathological outcomes. The principal finding is that the base-first approach consistently ensured inclusion of the mesoappendix, whereas the tip-first approach did not. While overall demographic and perioperative outcomes were similar between groups, the absence of mesoappendix in tip-first specimens posed a significant limitation for oncological staging when incidental malignancies were identified.

Our results highlight a pattern of surgical practice. Consultant surgeons more often employed the base-first technique, whereas the tip-first technique was more frequently used in non-consultant-led cases. This likely reflects differences in surgical experience and training exposure. Operative time did not differ significantly between the two techniques, indicating that removal of the mesoappendix does not add to procedure duration. Previous studies have described considerable variation in appendicectomy technique but have not classified these approaches explicitly [6-8]. By introducing this framework, our study provides a platform for standardisation in operative training.

Pathological findings carry the most important implications. In our series, incidental neoplasms were identified in 3.7% of appendicectomy specimens, consistent with previously reported rates of 1%-2% [9,10]. The histological subtypes included adenocarcinoma, neuroendocrine tumour, and goblet cell adenocarcinoma. In both malignancy cases managed with the tip-first technique, omission of the mesoappendix necessitated completion of a right hemicolectomy to enable adequate staging. Similar concerns have been raised by other authors who emphasised the importance of mesoappendix excision for accurate pathological assessment [11,12]. The mesoappendix contains lymphovascular channels, and failure to include it risks under-staging neoplasms, potentially altering long-term oncological outcomes [13-17]. Our findings therefore reinforce the oncological necessity of incorporating the mesoappendix in routine appendicectomy.

Operative and clinical outcomes such as operating time, length of stay, and complication rates were comparable between groups, aligning with established literature showing that technical modifications rarely alter these short-term endpoints [18]. The difference lies instead in the pathological completeness of the specimen. This distinction underlines why operative training should prioritise not only efficiency and safety but also diagnostic adequacy.

The findings of this study have direct educational implications. Given that tip-first resections were more common among junior surgeons, structured training programmes should emphasise the base-first technique [19]. Documenting the method of appendicectomy in operative notes may also assist pathologists in interpreting specimens and guiding follow-up care. Establishing base-first appendicectomy as the default approach would harmonise practice, safeguard oncological completeness, and reduce the need for secondary resections. This emphasis on standardisation is consistent with broader surgical training curricula and existing guideline principles, which prioritise both patient safety and oncological completeness.

Several limitations must be acknowledged. First, the retrospective and single-centre design introduces potential bias and limits generalisability. Second, the relatively small number of incidental neoplasms reduces the statistical power to evaluate oncological outcomes comprehensively. Third, the operative technique was inferred from histopathology rather than prospectively recorded, introducing a potential risk of misclassification. Fourth, variation in surgeon seniority and case severity (simple versus complicated appendicitis) may have acted as confounders, although no systematic differences were observed. Despite these limitations, the study contributes novel insight by explicitly defining and comparing base-first and tip-first techniques, an area previously overlooked in the literature.

In summary, our study demonstrates that while perioperative outcomes are similar between base-first and tip-first appendicectomy, only the base-first technique consistently ensures inclusion of the mesoappendix, which is critical for pathological staging. Standardising this approach within surgical training would enhance oncological safety and reduce the likelihood of further surgery when malignancy is encountered. Future prospective multicentre studies are warranted to validate these findings and to inform the incorporation of the appendicectomy technique into guideline development.

Beyond the immediate clinical implications, our findings should also be considered in the broader context of evolving guidelines and international literature. Several systematic reviews and guideline statements have highlighted the variability in appendicectomy techniques and emphasised the importance of standardisation to improve both short- and long-term outcomes [20,21]. Furthermore, studies investigating laparoscopic versus open approaches consistently demonstrate the role of surgical technique in shaping complication rates, even if perioperative outcomes remain broadly similar [22,23]. The oncological dimension of appendiceal pathology has also gained increasing attention, with consensus classifications and staging systems underscoring the importance of complete mesoappendix excision for accurate risk stratification [24-26]. More recent cohort and registry-based studies continue to reveal incidental neoplasms at clinically significant rates, reiterating the need for vigilance in specimen handling [27,28]. Finally, surgical education research strongly supports the incorporation of structured technical training and operative standardisation into curricula to reduce variability in care and optimise patient outcomes [29-30].

## Conclusions

This study demonstrates that while perioperative outcomes are comparable between base-first and tip-first appendicectomy, only the base-first technique consistently ensures inclusion of the mesoappendix, which is essential for complete pathological staging. In contrast, omission of the mesoappendix in tip-first specimens compromised oncological assessment in two cases, both of which required completion of right hemicolectomy.

By ensuring a diagnostically complete specimen without increasing operative risk, the base-first approach offers greater oncological safety. From an educational perspective, surgical training should emphasise the base-first technique, and documenting the operative method in notes may enhance communication with pathologists and guide follow-up care.

Although this retrospective single-centre study with a limited number of malignant cases cannot provide definitive recommendations, it introduces a clear classification framework for appendicectomy technique that has not, to our knowledge, been previously described. Prospective multicentre studies are warranted to validate these findings and to inform the incorporation of the appendicectomy technique into future guideline development.
